# Urétéroscopie souple laser dans le traitement des calculs du haut appareil urinaire: résultats a propos de 166 interventions

**DOI:** 10.11604/pamj.2015.22.13.7591

**Published:** 2015-09-08

**Authors:** Essodina Padja, Venceslas Amboulou Ibarra, Khalid Lmezguidi, Abdellatif Janane, Mohamed Ghadouane, Ahmed Ameur, Mohamed Abbar

**Affiliations:** 1 Service d'Urologie de l'Hôpital Militaire d'Instruction Mohammed V de Rabat, Université Mohammed V-Rabat, Maroc

**Keywords:** calculs, haut appareil urinaire, urétéroscopie souple, lithotripsie par laser, résultats, kidney stones, upper urinary tract, flexible ureteroscopy, lithotripsy laser, result

## Abstract

L’évolution de la technique opératoire fait de l'urétéroscopie souple –Laser (URSS-L) une méthode efficace et sure dans le traitement des calculs du haut appareil urinaire (HAU). Elle apparait comme une option salvatrice après échec des autres options thérapeutiques. Son coût limite son accessibilité et restreint ses indications dans certains contextes socio-économiques. Nous rapportons l'expérience du service sur les indications, les résultats et les complications de l'URSS-L dans le traitement des calculs du HAU. C'est une étude rétrospective sur 4ans concernant 130 patients ayant des calculs du HAU. Un scanner permettait de déterminer les caractéristiques des calculs avant l'intervention et de faire un contrôle après afin de juger de l'efficacité du traitement. Une analyse statistique évaluait l'influence des différents paramètres des calculs sur l'efficacité de l'intervention. Un suivi était réalisé afin de détecter les complications. L’âge moyen des patients était de 52 ± 17ans. 166 interventions étaient faites en 3 séries. Les indications étaient de première intention dans 50.32% suivi des échecs de LEC. La durée moyenne de l'intervention était de 73min ± 25min pour une taille moyenne des calculs de 13.78mm ± 5mm. Le taux de succès global était de 78.91% (78.71%; 80%; 100%) respectivement après la 1ère, la 2ème et la 3ème série. Aucun paramètre n'influençait significativement le taux de succès. 14.45% de complications était enregistré. L'URSS-L est une méthode aussi efficace que sure dans le traitement des calculs du HAU nous motivant malgré son coût, à élargir ses indications en première intention lorsque les calculs répondent aux critères de choix.

## Introduction

L'apparition des urétéroscopes de 2^ème^ génération et l’évolution de la technique opératoire font de l'urétéroscopie souple-Laser (URSS-L) une méthode efficace et sure dans le traitement des calculs du haut appareil urinaire (HAU). Le choix thérapeutique dans le traitement des calculs du HAU dépend de plusieurs paramètres (facteurs de comorbidité associés,symptômes, localisation, taille, nature biochimique des calculs). L'URSS-L parait être le traitement adéquat pour les calculs caliciels inférieurs de moins de 2cm à cause du faible taux de succès de la LEC [1]; dans d'autres situations comme après un échec de la lithotripsie extracorporelle (LEC) ou la persistance de fragments résiduels après une Néphrolithotomie percutanée (NLPC), l'URSS-L se distingue comme une méthode salvatrice dans le traitement de ce type de calculs. Sa faible morbidité pousse certains urologues à préférer plusieurs séances d'URSS-L à une NLPC lorsque la taille des calculs dépasse 20mm [2]. Mais son coût élevé et la fragilité du matériel limite son accessibilité et restreint ses indications dans certains contextes socio-économiques comme le nôtre. Dans ce contexte nous rapportons à travers 166 interventions notre expérience sur les indications les résultats et les complications de l'URSS-L dans le traitement des calculs du HAU.

## Méthodes

Notre étude était rétrospective concernant 130 patients (79 hommes et 51 femmes) sur une durée de 04 ans (d'août 2010 à octobre 2014). L'intervention a concerné 155 calculs rénaux et urétéraux proximaux. Pour tous nos patients, un uroscanner ou un scanner abdominopelvien (TDM AP) non injecté était réalisé afin de déterminer les caractéristiques des calculs (taille, emplacement, densité et nombre). Un examen cytobactériologique des urines (ECBU) stérile datant de moins de 3 semaines avant l'intervention était obligatoire. Les interventions étaient faites sous anesthésie générale par deux chirurgiens; au total 166 ont été réalisées. L'administration d'une antibiothérapie prophylactique à base de céfazoline 1g était systématique. L'appareil laser utilisé était le MEGA Pulse délivrant une puissance comprise entre 1.6W et 20 W. En fonction de la nature du calcul nous utilisions une puissance de 3 à 15W. Pour les calculs (urétéraux, pyéliques et caliciel supérieur) ne nécessitant pas une déflexion importante de l'urétéroscope, une fibre de 365µm était utilisée. Pour les localisations nécessitant une déflexion importante comme les calculs caliciels inférieurs, une fibre de 230µm. Nous avons utilisé systématiquement des gaines d'accès de 35 ou 45 cm de longueur avec un diamètre externe de 14ch et interne de 12ch. Dans la plupart des cas le calcul était vaporisé mais il arrivait que nous obtenions des petits fragments qui étaient récupérés par une pince à panier type Dormia ou une N-Gage dans 42.16% des cas (n = 70). Au cours de notre expérience nous avons eu 2 urétéroscopes cassés et un avait pu être réparé. Les résultats, l'influence des différents paramètres des calculs sur les résultats et les complications des interventions ont été évalués. L'analyse statistique était faite par le logiciel SPSS.20 (différence significative lorsque P ≤ 0.01). Un ASP (cliché d'abdomen sans préparation) et ou une échographie et ou une TDM AP étaient réalisés pour évaluer l'efficacité de l'URSS-L. Le succès était défini par l'absence de fragments ou la présence de fragments de moins de 3mm sur l'imagerie de contrôle.

## Résultats

Les données des patients et les caractéristiques des calculs sont mentionnées dans le Tableau 1. L’âge moyen des patients était de 52 ± 17ans (14-76 ans); 51 femmes et 79 hommes. L’étiologie des calculs était le plus souvent indéterminée. 166 interventions ont été réalisées durant cette période en 3 séries (dont 155 durant la 1^ère^, 10 durant la 2^ème^ et 1 durant la 3^ème^). Les indications de l'URSS-L étaient de première intention dans 50.32% des cas suivie des échecs de LEC dans 17,41% des cas. Les critères pour l'indication de première intention étaient les suivent: patients avec trouble de la crase sanguine ou sous traitement anticoagulant, localisation calicielle inférieure des calculs, malformation rénale ou rein unique, obésité, densité du calcul largement au-dessus de 1000UH. Le Tableau 2 résume les différentes indications de l'URSS-L. La durée moyenne des interventions était de 73min ± 25min (30 min -135min) avec une taille moyenne des calculs de 13.78mm±5mm (6-20mm). Dans trois cas, nous avons eu des incidents per-opératoires obligeant l'arrêt de l'intervention: 2 cas d'hématurie importante empêchant une bonne visibilité dont une secondaire à une perforation urétérale ayant nécessité une pose de sonde JJ et la reprise des patients après 2 et 4 semaines; Un cas de pyurie franche dans un groupe caliciel malgré un ECBU préopératoire stérile. Ceci a nécessité une pose d'une sonde JJ, une antibiothérapie et la reprise du patient après 4 semaines. Sur les 166 URSS-L réalisées, nous avons: effectué un drainage par une sonde urétérale dans 18.68% des cas (n = 31); Effectué un drainage par une sonde double JJ dans 30.12% des cas (n = 50); Opté pour l'absence de drainage dans 51.20% des cas (n = 85). La durée moyenne d'hospitalisation était de 2 jours (1.5 jours - 6 jours). Tous les patients ayant plus de 2 jours d'hospitalisation étaient des patients qui avaient des complications en post opératoire immédiat (hématurie, douleurs ou pyélonéphrite aigue) L'imagerie de contrôle était réalisée entre 1 et 4 mois après l'intervention. Le taux global de succès (défini comme une absence de fragments ou la présence de fragments de moins de 3 mm) était de 78.91% (78.71%; 80%; 100%) respectivement après la 1ère, la 2ème et la 3ème série d'URSS-L. Les données d'analyse uni variée déterminant l'impact des paramètres des calculs sur le succès de l'URSS-L sont résumées dans le Tableau 3. Le taux de succès était plus bas lorsque la densité des calculs >1000UH, le calcul était au niveau caliciel inférieur ou encore lorsque le calcul avait une taille > 15mm. Au final malgré ces variations, aucun paramètre n'influençait significativement le taux de succès. Peu de complications ont été enregistrées (14.45%): 1 cas de perforation urétérale, 10 cas de PNA ayant évolué favorablement sous traitement antibiotique adapté. Sur les 10 cas de PNA, 4 sont survenues en post opératoire précoce (moins d'une semaine après l'intervention) et les 6 autres entre 7 et 21 jours après l'intervention. Parmi les autres complications, 8 cas de douleurs lombaires ayant bien évolué sous traitement symptomatique; 4 cas d'hématurie macroscopique résolues spontanément; et 1cas de sténose uretero-pyélique survenue tardivement et traitée par endopyélotomie ont été rapportés. Nous n'avons enregistré aucune augmentation du taux de créatinine sanguin en post opératoire immédiat, précoce ou tardif chez nos patients. Selon la classification de Clavien-Dindo, les complications étaient de grade I dans 7.22% (soit 50% des complications); de grade II dans 6.02% (41.67% des complications) et de grade III dans 1.2% (8.33% des complications). Aucune complication de grade IV ou V n'a été enregistrée. L'ensemble de ces complications sont résumées dans le Tableau 4.

**Table 1:** Données démographiques des patients et caractéristiques des calculs

	Nombre	Pourcentage (%)
Caractéristiques des patients		
Age		
	Moyenne 52 ±17ans	
	Min: 14ans	
	Max: 76ans	
Hommes	79	60.77
Femmes	51	39.23
Caractéristiques des calculs		
Latéralité		
Droite	62	47.70
Gauche	53	40.77
Bilatérale	25	19.23
Localisation du calcul		
Uretère (proximal)	26	16.77
Calice supérieur	39	25.16
Calice moyen	34	21.94
Calice inferieur	56	36.3
Densité du calcul (UH)		
Moyenne 985.48		
D ≤ 500U	30	19.35
500< D ≤ 1000	58	37.42
D > 1000	67	43.23
Taille du calcul (mm)		
Moyenne	13.78mm±5mm (6-20mm)	
T ≤ 10	33	21.29
10< T≤ 15	65	41.94
T > 15	57	36.77
Pathologies sous-jacentes		
Hyperparathyroïdie	7	5.38
Goûte	3	2.31
Oxalose primitive	1	0.77
Cystinurie	3	2.31
Indéterminées	116	89.23

**Table 2:** Indications de l'URSS-L

	Nombre	Pourcentage (%)
1^**ère**^ intention	78	50.32
Echec de LEC	27	17.42
Echec de traitement médical	20	12.90
Echec d'urétéroscopie rigide	15	9.68
Fragment résiduels après NLPC	15	9.68

**Table 3:** Taux de succès en fonction des variables

Taux de succès global	78.91%	
Après 1^**ère**^ série d'URSS-L	78.71%	
Après 2^ème^ série d'URSS-L	80%	
Après 3^ème^ série d'URSS-L	100%	
Taux de succès en fonction des différentes caractéristiques des calculs		
Caractéristiques des calculs	Taux de succès	Valeur de P
Localisation du calcul		
Urétérale (proximal)	96.15%	
Caliciel sup	79.49%	0.6
Caliciel moyen	79.41%	
Caliciel inf	71.42%	
Taille du calcul (mm)		
≤10	90.90%	0.8
10 < T ≤ 15	78.46%	
15 < T ≤ 20	71.93%	
Densité du calcul (UH)		
D ≤ 500	90%	
500 < D ≤ 1000	82.76%	0.1
D > 1000	70.14%	

**Table 4:** Complications avec la classification de Clavien- Dindo-Strasberg

Type de complications (grade Clavien –Dindo)	Nombre	Pourcentage (%)
PNA (grade II)	10	6.02
Douleurs lombaires ou coliques néphrétiques (grade I)	8	4.82
Hématurie (grade I)	4	2.41
Sténoses urétérales (grade IIIb)	1	0.6
Perforation urétérale (grade IIIb)	1	0.6

## Discussion

Depuis Dretler en 1994 [3] qui a décrit la technique d'urétéroscopie souple, celle-ci a connue d’énormes avancées technologiques notamment l'utilisation de la déflexion active à 270° qui a permis une exploration de l'ensemble des cavités rénales [4], permettant l’élargissement des indications. L'URS-SL est une approche moderne du traitement des calculs du rein et de l'uretère. De par sa nature endoscopique et parce que la lithotritie se déroule par vaporisation LASER holmium de contact, elle répond au traitement de tous les types de calcul; aucun calcul ne résistant au laser [5]. Les indications de l'URSS-L en première intention dans le traitement des calculs du haut appareil urinaire sont bien établies par Le comité de lithiase de l'AFU et d'autres sociétés savantes [6]. Dans la série de B. Fall et al [7]; les indications de 1^ère^ intention représentaient 62.3% par rapport à 50.32% dans notre série. Ceci peut s'expliquer par le coût élevé de cette modalité thérapeutique et la fragilité du matériel nous poussant à limiter la taille des calculs à 20mm et à privilégier d'autre modalités thérapeutiques comme la LEC ou la NLPC dans certaines situations. Plusieurs auteurs ont rapporté à travers leur expériences l'efficacité de l'URSS-L dans le traitement des calculs et notamment les calculs de moins de 2 cm de diamètre. En effet, E. Lechevalier et ses collaborateurs [8] rapportent un taux de succès global pour les calculs rénaux entre 65 et 85% et pour les calculs urétéraux entre 75 et 90%. Dans l’étude de P-O. FAIS, le taux de succès pour les calices supérieurs et le bassinet sont de 60 à 100%, et de 60 à 80% pour les calices inférieurs [9]. Quand à M.A. Ben Saddik et ses collaborateurs [2] qui se sont intéressés à des calculs de 2 à 3cm, leur taux de succès global était de 63,1, 89,3 et 97,1% respectivement après une, deux et trois séances d'URSS-laser. B.Fall et col rapportent dans leur série un taux de succès global de 71,7%. Nous avons eu un taux de succès global de 78.91% pour tous les calculs. Le taux de succès pour les calculs urétéraux dans notre série était de 96.15% par rapport à un taux de succès de 79.49%, 79.41% et 71.42% respectivement pour les calculs caliciels supérieurs, moyens et inférieurs. Ces résultats sont comparables à ceux de la littérature mais il faut souligner que la taille maximale des calculs dans notre étude était de 20mm et les calculs urétéraux étaient tous au niveau de l'uretère proximal. En analyse uni variée, aucun paramètre ne semblait influencer significativement le taux de succès dans notre étude. Le taux de succès pour les calculs caliciels inférieurs était bien inférieur à celui des autres calculs mais la différence n’était pas significative. Dans l’étude de B.Fall [7] l'expérience du chirurgien était le paramètre qui modifiait significativement les résultats de l'intervention. M.A. Ben saddik [2] rapportait une différence significative liée à la taille du calcul mais pour des calculs de 20-30mm. Un faible taux de morbidité se greffe à l'URSS-L dans le traitement des calculs rénaux et urétéraux. En effets beaucoup d’étude récentes réalisées à son sujet rapportent très peu de complications et n'ont pas mis en évidence de complications majeures [10]. La littérature rapporte une morbidité globale de l'urétéroscopie de 5-10% [8]. Le risque de complication majeure (avulsion, perforation) est de 1%. Le risque de complication tardive est dû aux sténoses et est de l'ordre de 1%. Le risque d'infection fébrile après urétéroscopie est de 2-18% [8]. Dans notre étude nous avons eu un taux de complication global de 14.46% dont la majorité (41.67%) était d'origine infectieuse avec une bonne évolution sous traitement antibiotique; un seul cas (0.6%) de complication grave de type perforation urétérale et à la longue cette perforation ne s'est pas compliquée de sténose. Dans les suites tardives, un seul cas (0.6%) de sténose urétérale. Ces taux de complications sont comparables à ceux de la littérature et viennent conforter l'idée selon laquelle l'URSS-L est une méthode greffée de très peu de morbidité. Concernant le drainage post opératoire, aucune étude n'a lié l'absence ou le type de drainage poste opératoire à la survenue des complications; pour l'heure, il n'y a pas de consensus sur l'omission ou le type de drainage à réaliser en post opératoire. Les données en faveur d'un drainage post opératoire sont: un calcul impacté, une longue durée d'intervention, lésion de la muqueuse urétérale lors de l'intervention, présence de fragments après l'intervention, l'appréciation et la tendance de l'opérateur. Dans notre série, nous avons recouru au drainage dans 48.8% des cas. Quant à la durée de l'intervention, elle est fonction des paramètres du calcul (taille, localisation, densité), de la qualité de l'urétéroscope pour une bonne visibilité, du choix adéquat des paramètres du laser en fonction de la nature du calcul mais aussi et surtout de l'expérience de l'opérateur. Dans notre étude la durée moyenne de l'intervention était de 73min ± 25min pour une taille moyenne de 13.78mm ± 5mm. Les durées rapportées dans la littérature sont extrêmement variables mais il faut en général 60min pour fragmenter un calcul de 10mm.

## Conclusion

Notre étude comme celles déjà publiées vient montrer que l'URSS-L est une méthode aussi efficace que sure dans le traitement des calculs rénaux et urétéraux. Malgré son coût, l'obtention de bons résultats et sa faible morbidité nous motive à élargir ses indications en première intention lorsque les calculs répondent aux critères de choix.
